# Readiness to Be Physically Active and Self-Reported Physical Activity in Low-Income Latinas, California WISEWOMAN, 2006-2007

**Published:** 2012-04-19

**Authors:** Karen J. Coleman, Maureen A. Farrell, David A. Rocha, Toshi Hayashi, Marianne Hernandez, Janet Wolf, Sue Lindsay

**Affiliations:** Southern California Permanente Medical Group, Research and Evaluation; California Department of Public Health, Sacramento, California; California Department of Public Health, Sacramento, California; California Department of Public Health, Sacramento, California; California Department of Public Health, Sacramento, California; San Diego State University, San Diego, California; San Diego State University, San Diego, California

## Abstract

**Introduction:**

Latinas are more likely to be inactive than non-Hispanic white women. Although 74% of Latinas report no leisure-time activity, few interventions have been designed to promote physical activity among these women. The objective of this study was to assess the effect of the California WISEWOMAN program on low-income Latinas's readiness to change physical activity and on self-reported physical activity behaviors.

**Methods:**

We screened 1,332 women for cardiovascular disease risk factors and randomly assigned 1,093 women to 2 groups: an enhanced intervention (n = 552) or usual care (n = 541). The enhanced intervention was delivered by community health workers in one-on-one counseling sessions. We examined self-reported readiness to change and physical activity at baseline and 12-month follow-up among participants who completed both assessments (n = 868).

**Results:**

Mean age of participants was 52 years (standard deviation, 6 y); most (65%) were Mexican or Mexican American, and most (81%) were not high school graduates. A higher percentage (67%) of the enhanced intervention group was in the action/maintenance stage for vigorous physical activity at follow-up compared with baseline (47%). We found no such change among women in usual care (52%, baseline; 58%, follow-up). A higher percentage of the enhanced intervention group also reported significant increases in moderate (71%, baseline; 84%, follow-up) and vigorous (13% to 33%) physical activity at follow-up than at baseline. Women in usual care reported no changes.

**Conclusion:**

A culturally tailored adaptation of the WISEWOMAN program that used community health workers significantly improved both self-reported readiness to engage in physical activity and vigorous physical activity among low-income Latinas.

## Introduction

Although death from cardiovascular disease (CVD) has declined in recent decades, disparities exist for low-income Latinas (1,2). These disparities may be due to high rates of obesity and low levels of physical activity. Latinas are more likely to be inactive than non-Hispanic white women (2); 74% of Latinas report no physically active leisure-time activity (3). Despite the obvious public health need, few interventions have been designed to promote physical activity among Latinas. A recent review of the literature found only 12 intervention studies that promoted physical activity in Latinas; only 4 showed significant increases in physical activity, and few had any long-term follow-up to determine maintenance of physical activity changes (4). Many of these interventions may have failed because they had a poor foundation in behavior-change theory, especially as the theory relates to the cultural and contextual factors that determine behavior in Latinas (4).

To address this limitation, the California Department of Public Health (CDPH) tested a cultural adaptation for Latinas of a successful program used to promote healthy behaviors in African American women who have CVD (5-7). The program was created by the University of North Carolina at Chapel Hill (UNC-Chapel Hill) for use by the North Carolina Well-Integrated Screening and Evaluation for Women Across the Nation (WISEWOMAN) program (8). Behavior-change theory, especially as interpreted through the transtheoretical and socioecological models (9), was a critical foundation of the original program and of our adaptation. All aspects of the California program were delivered by bicultural, bilingual community health workers (CHWs) (8). The program resulted in improvements in the CVD risk profile, as measured by the 10-year coronary heart disease risk, of women who participated in the intervention group compared with women in usual care (10).

The objective of this study was to assess the effect of the California WISEWOMAN program on participants'readiness to change 4 physical activity behaviors (engaging in vigorous physical activity, taking up new physical activities, doing daily activities more briskly, and changing daily habits to be more active) and self-reported physical activity. We measured readiness to change to verify the theoretical foundations of the intervention and to study how changing readiness to engage in behavior was related to self-reported behavior change.

## Methods

### Study design

Study design was a within-site randomized intervention of 2 study groups (enhanced intervention group [EIG] and usual care group [UCG]) from January 2006 to June 2007. Details on study design, eligibility, recruitment, enrollment, and initial study outcomes are described elsewhere (8,10). The California WISEWOMAN study protocol was approved by the Centers for Disease Control and Prevention (CDC) and the California Committee for the Protection of Human Subjects institutional review boards.

### Study settings

Sites for both the EIG and UCG were community clinics that were participating in the California National Breast and Cervical Cancer Early Detection Program (NBCCEDP) and serving a large population of low-income, Latino patients. We selected the 4 clinics (2 in San Diego and 2 in Los Angeles counties) in 2005 through a competitive application process. The mission of these clinics was to address the health concerns of primarily Hispanic/Latino low-income communities; health care providers spoke both Spanish and English.

### Eligibility, recruitment, and enrollment

We screened 1,332 Latinas for eligibility; 1,093 women met the inclusion criteria (8,10). Women were recruited through the NBCCEDP by telephone calls, cards and letters, and face-to-face invitations during NBCCEDP appointments. To be eligible, women were required to self-identify as Hispanic or Latina, speak English or Spanish, be aged 40 to 64 years, have an annual income at or below 200% of the federal poverty level, have inadequate or no health insurance, have a blood pressure or total cholesterol level considered at risk for developing CVD (either ≥120 mm Hg systolic or ≥80 mm Hg diastolic for blood pressure or currently taking medications to lower blood pressure and either ≥200 mg/dL for total cholesterol or taking medications to lower cholesterol). Women were excluded if they were pregnant or planning to become pregnant during the study period, if they had current or past history of a CVD event or condition, or if they had a blood pressure measurement of 180/110 mm Hg or more or a cholesterol measurement of 400 mg/dL or more. Women with these high values were immediately referred for follow-up care. Eligibility was determined by a nurse. After baseline assessment and clinical measurements, the clinic study team nurse randomly assigned eligible participants to the EIG (n = 552) or UCG (n = 541). We enrolled participants between January 2006 and August 2006.

### Intervention

**Enhanced intervention.** The enhanced intervention was delivered by CHWs as 3 individually tailored, one-on-one counseling sessions. The sessions occurred approximately 1 month, 2 months, and 6 months after screening. Each session averaged 50 minutes. Participants received transportation tokens and grocery store vouchers as incentives for attendance.

Adaptation of the WISEWOMAN curriculum for our participants is detailed elsewhere (11) and followed expert recommendations (12). In short, to adapt the program, investigators at UNC-Chapel Hill supervised an expert panel review, conducted focus groups, translated the program to Spanish, elicited feedback on the Spanish version, back-translated the new version to English, and reviewed the back-translation to ensure fidelity to the original program. *Vida Saludable, Corazón Contento!* is available from the Center of Excellence for Training and Research Translation (5).

The curriculum emphasized 6 core elements: assessment, tailored feedback, goal setting, guidelines and strategies to overcome barriers, follow-up and reinforcement, and social support. It also used theoretical concepts, such as self-efficacy, self-regulation through self-monitoring, and readiness for change. We tailored the program for each participant by using their self-identified barriers and facilitators to behavior change and emphasizing contextual factors important to Latinas, such as family and social support (4). We also allowed women to choose different activities to meet their goals as long as they were at least of moderate intensity.

We visited each study clinic site twice during the study period to ensure fidelity to the study protocol. We determined whether all program elements were present, observed screening and counseling sessions, assessed accuracy of medical record chart abstraction, and held discussions with site staff and administrators. We used monthly teleconferences for similar purposes. Because CHWs delivered the enhanced intervention and collected data on self-reported behavior, we emphasized the importance of avoiding bias in data collection.

**Usual care.** UCG participants received usual care for elevated blood pressure or cholesterol from each clinic site. EIG participants had the same access to usual care. Usual care may have included any or all of the following: healthy behavior education by a provider, distribution of healthy lifestyle handouts, and referral of women to healthy lifestyle education classes if available at the clinic site. Care was provided by physicians and nurses in Spanish and English at each clinic and consisted of monitoring blood pressure and cholesterol and any medications taken by the patients, addressing any other patient health concerns, and performing routine health screenings. UCG participants also received incentives during the baseline and follow-up assessments.

### Community health workers

The CHWs hired to deliver the program were of the same communities as the study sites and were required to be bilingual (in Spanish and English) and bicultural (have a similar heritage to program participants), have direct experience with Spanish-speaking immigrant communities, and have basic computer skills. Of the 8 CHWs hired, 7 were younger than 30 years, 6 had at least 2 years of college, and 2 had obtained postgraduate degrees in Mexico. All were women. All were paid salaries by the clinics, which used funds from the CDPH through a CDC grant. All CHWs remained employed by the clinics during the intervention and follow-up. Before the study began, CHWs received an intensive 2½ days of initial training on how to deliver the enhanced intervention; the training was repeated 3 months into the study (8). The training included information on how to administer study protocols, conduct nutrition and physical activity behavior-change counseling, and collect self-reported data. In addition, CHWs and study staff participated in monthly 1-hour teleconference meetings. Training was provided by the California WISEWOMAN program staff and other state program partners.

### Measurements

**Assessments. **We assessed readiness to change behavior and self-reported physical activity by using 2 separate questionnaires during individual appointments 1 week after a woman's enrollment. Women began the intervention 1 month after their baseline assessment. Follow-up assessment was scheduled by appointment between 9 and 14 months after a woman's baseline assessment, and the average time to follow-up was 12 months. Of the 1,093 women selected to participate, 868 (79%) completed both the baseline and follow-up assessments. We were unable to contact the remaining 225 women (119 women in the EIG and 106 women in the UCG). The EIG and UCG at baseline did not differ in medication use, blood pressure, cholesterol, obesity rates, or 10-year coronary artery disease risk (10).

**Readiness for behavior change.** At baseline and follow-up, CHWs administered a questionnaire to all participants on readiness to change 4 behaviors: 1) engaging in physical activity “hard enough to increase your breathing for at least 30 minutes 4 times a week,” which we defined as vigorous activity; 2) taking up new physical activities; 3) adding intensity to daily activities (ie, performing normal activities such as vacuuming or gardening); and 4) changing daily habits to increase activity (eg, taking stairs instead of elevator). Possible responses to each question were, “No, I haven’t thought about being more physically active and have no plans to change”; “No, but I am thinking about doing this sometime in the next 6 months”; “No, but I plan to start doing this sometime in the next month”; “Yes, I do this now, but it has been less than 6 months since I started”; “Yes, I do this now and have been doing this for 6 months or longer.” We recoded these 5 possible responses into 2 variables: precontemplation/contemplation/preparation and action/maintenance. The format and design of the questionnaire were based on the work of Marcus and Simkin (13) and physical activity stages-of-change questionnaires adapted for low-income women (14) and low-income Mexican women (15). The stages-of-change model has good predictive validity for physical activity in low-income Mexican women in community settings (15) and low-income African American women in clinical settings (16). Cronbach α for the Spanish version of the questionnaire used in our study was α = 0.65.

**Self-reported physical activity.** We measured self-reported physical activity at baseline and follow-up by using 2 items on the 8-item Spanish version of the Physical Activity Assessment survey developed by UNC-Chapel Hill (5,9) as part of the North Carolina WISEWOMAN program. We asked participants about their participation in walking or running by using the question, "Do you walk or run (for fun, exercise, transportation)?" We asked about participation in exercise or sports by using the question, "Do you do either of these [sports or exercise]?" We considered walking or running moderate physical activity and exercise or sports vigorous physical activity. The categories of intensity were validated against accelerometry (17). The answer for each question was yes or no. The Spanish version was adapted and translated with the curriculum as detailed earlier.

### Analyses

We used data only from participants who completed both the baseline and follow-up assessments. We analyzed both dichotomous variables (stage of change and self-reported physical activity) in separate intent-to-treat mixed models using SAS version 9.1.3 (SAS Institute Inc, Cary, North Carolina) to account for the correlation within subjects of responses across time (18). The associations are expressed as odds ratios (ORs) and 95% confidence intervals (CIs). We examined post-hoc differences for interaction effects using the McNemar test for repeated measures and the χ^2^ test for group differences.

## Results

### Participant characteristics

The demographic characteristics of the EIG and UCG did not differ significantly at baseline (Table 1). Women who self-reported data for both assessments did not differ in education from women had only a baseline assessment; however, they were more likely to identify as Mexican or Mexican American than women who had only a baseline assessment.

### Self-reported readiness for change

Women in the EIG and UCG did not differ significantly at baseline in self-reported readiness to change any of the 4 physical activity behaviors assessed (Table 2). The EIG was twice as likely to report being in the action/maintenance stage for vigorous physical activity at follow-up than at baseline; 47% of women in this group reported readiness to engage in vigorous physical activity at baseline, and 67% at follow-up. The UCG's readiness for vigorous physical activity did not change between baseline and follow-up; 52% of women in this group reported readiness at baseline, and 58% at follow-up. Both groups reported significant increases in readiness to take up new physical activity, perform daily habits more briskly, and incorporate physical activity into daily activity. The increases were greater for the EIG than for the UCG.

### Self-reported physical activity

The EIG and UCG did not differ significantly at baseline in either category of self-reported physical activity (Table 3). The EIG was approximately twice as likely to report engaging in moderate physical activity at follow-up as at baseline; 71% of women in this group reported moderate physical activity at baseline, and 84% at follow-up. The UCG did not change between baseline and follow-up; 75% of women in this group reported moderate physical activity at baseline, and 77% at follow-up. The EIG was approximately 3 times as likely to report engaging in vigorous physical activity at follow-up as at baseline; 13% of these women reported moderate activity at baseline, and 33% at follow-up. Women in the UC group showed no change (16%, baseline vs 17%, follow-up).

## Discussion

Only women in the EIG self-reported significant increases in both moderate and vigorous physical activity. More women in both groups at follow-up than at baseline reported being in the action/maintenance stages of readiness for 3 of the 4 physical activity behaviors. The change was greater for the EIG than for the UCG. Only the women in the EIG changed their readiness to engage in vigorous physical activity.

Our findings support other studies showing that an increase in a person's readiness to change through health education alone often does not lead to changes in behavior (19). Health education is only 1 element of a comprehensive strategy for changing physical activity behavior, which also includes social support (20), reliance on self to overcome barriers (21), and restructuring the physical environment (22). These were all elements of the California WISEWOMAN intervention. In addition, research supports the addition of CHWs to health promotion research teams (23). CHWs help with recruitment (24) and retention (25) and may be more effective interventionists than traditional research staff (23).

Our findings on changes in self-reported moderate and vigorous physical activity are similar to the findings of 4 studies that found significant increases in physical activity among Latinas (26-29). Most interventions for promoting physical activity among Latinas have intensive components such as exercise classes and walking several times a week for 2 to 10 months (29,30), frequent staff contact and counseling (26), and small sample sizes. It would be difficult to implement such interventions in clinical settings that have limited resources or provide services to thousands of patients annually. Our minimal-contact intervention (2 assessments and 3 behavior-change counseling visits) is much better suited to a variety of clinical settings that serve large numbers of low-income Hispanic/Latino patients who are at high risk for CVD.

Our study had several limitations. We did not objectively measure physical activity as other studies have done (7,17). People over-report self-reported physical activity relative to physical activity measured objectively by activity monitors (31). However, self-report still provides more detail than activity monitors on the activities in which participants engage; activity monitors do not provide a comprehensive understanding of physical activity behavior (32). Our self-reported data is supported by previously reported results of the California WISEWOMAN intervention: women in the EIG reduced their 10-year coronary heart disease risk relative to women in the UCG (10).

Another limitation is that we did not standardize the information or treatment of women in the UCG. Some of these women may have received information and attended classes on healthy lifestyles during the intervention. All women in our intervention were enrolled in the California NBCCEDP, so all may have received healthy lifestyle information through this program or another community program. These exposures are not likely to have accounted for the effect of our intervention, however, because all participants were equally exposed. A third limitation is that follow-up data were collected by the same CHWs who delivered the intervention. Although CHWs were trained to be aware of and avoid bias when collecting follow-up data, our findings would have been strengthened had we had different follow-up data collectors.

Despite these limitations, our study has a number of strengths. One was the large sample size. Many published WISEWOMAN intervention studies had only one-quarter to one-half the sample size of our study (5-9,9,19). In addition, our study was conducted with strong design characteristics, such as randomization and standardization of the intervention protocol. We used the CHW model of health promotion, which is designed for community clinics that serve large populations of immigrant, non-English–speaking, low-income patients. Several studies have demonstrated the effectiveness of the CHW model for promoting health behaviors in community settings (23-25); ours is one of the first to demonstrate their important contribution to health promotion in a clinical setting. The California WISEWOMAN program is ideal for health promotion in clinical settings that serve low-income immigrant communities at high risk for CVD because of its strong foundation in behavior-change theory, its commitment to the CHW model, and its emphasis on initiating more vigorous physical activity.

## Figures and Tables

**Table 1.** Baseline Characteristics of Participants Who Completed Assessment at Baseline and 12-Month Follow-Up (N = 868), by Study Group, WISEWOMAN Program California, 2006-2007^a^

**Characteristic**	Enhanced Intervention Group (n = 433)	Usual Care Group (n = 435)	Overall (n = 868)
**Preferred language**			
Spanish	427 (99)	432 (99)	859 (99)
English	6 (1)	3 (1)	9 (1)
**Age, mean (SD), y**	52 (6)	52 (6)	52 (6)
**Ethnicity**			
Mexican or Mexican American	285 (66)	283 (65)	568 (65)
Central or South American	148 (34)	152 (35)	300 (35)
**Level of education**			
<High school	317 (73)	301 (69)	618 (71)
Some high school	39 (9)	46 (11)	85 (10)
High school graduate	56 (13)	57 (13)	113 (13)
Some college	21 (5)	31 (7)	52 (6)

Abbreviation: SD, standard deviation.

a Values are numbers (percentages) unless otherwise indicated. Using χ^2^analyses for proportional data and independent samples *t* tests for continuous data, we found no significant differences between the 2 study groups.

**Table 2.** Participants Who Self-Reported Being in the Action/Maintenance Stage of Readiness for Change at Baseline or 12-Month Follow-up (N = 868), by Study Group, WISEWOMAN Program, California, 2006-2007^a^

Behavior^b^	Enhanced Intervention Group (n = 433)			Usual Care Group (n = 435)		

	Baseline, n (%)	Follow-up, n (%)	OR (95% CI)	Baseline, n (%)	Follow-up, n (%)	OR (95% CI)
Engage in vigorous physical activity^c^	205 (47)	291 (67)	2.34 (1.77-3.09)	227 (52)	251 (58)	1.26 (0.96-1.65)
Take up new physical activity	98 (23)	244 (56)	4.53 (3.37-6.10)	132 (30)	212 (49)	2.22 (1.68-2.95)
Perform daily activities more briskly	135 (31)	288 (67)	4.52 (3.39-6.02)	164 (38)	268 (62)	2.72 (2.06-3.59)
Incorporate physical activity into daily activity	198 (46)	350 (81)	5.21 (3.82-7.09)	204 (47)	315 (72)	3.06 (2.30-4.08)

Abbreviations: OR, odds ratio; CI, confidence interval.

a BMI (kg/m^2^) categories defined for all races/ethnicities by the National Heart, Lung, and Blood Institute (15) as follows: underweight, <18.5; normal weight, 18.5-24.9; overweight, 25.0-29.9; class I obese, 30.0-34.9; class II obese, 35.0-39.9; and class III obese, ≥40.0.

b Behavior categories are not mutually exclusive; participants were asked about readiness to change in all 4 behavior-change categories.

c Vigorous activity defined as physical activity hard enough to increase breathing for at least 30 minutes 4 times per week.

**Table 3.** Participants Who Self-Reported Moderate and Vigorous Physical Activity at Baseline or 12-Month Follow-up, by Study Group, WISEWOMAN Program, California, 2006-2007

Behavior^a^	Enhanced Intervention Group (n = 433)				Usual Care Group (n = 435)			

	Baseline, n (%)	Follow-up, n (%)	OR (95% CI)	*P*	Baseline, n (%)	Follow-up, n (%)	OR (95%CI)	*P*
Moderate	309 (71)	365 (84)	2.19 (1.57-3.07)	<.001	328 (75)	335 (77)	1.10 (0.80-1.50)	.57
Vigorous	57 (13)	143 (33)	3.37 (2.38-4.77)	<.001	69 (16)	75 (17)	1.11 (0.77-1.59)	.58

Abbreviations: OR, odds ratio; CI, confidence interval.

a Moderate activity defined in the questionnaire as walking or running. We asked participants about their participation in walking or running by using the question, "Do you walk or run (for fun, exercise, transportation)?" Answer was yes or no. Vigorous activity defined as exercise or sports. We asked about participation in exercise or sports by using the question, "Do you do either of these [sports or exercise]?" Answer was yes or no. Dichotomized values were analyzed by using intent-to-treat mixed models.
